# Impact of patient comfort on diagnostic image quality during PET/MR exam: A quantitative survey study for clinical workflow management

**DOI:** 10.1002/acm2.12664

**Published:** 2019-06-17

**Authors:** Shuguang Chen, Pengcheng Hu, Yusen Gu, Lifang Pang, Zheng Zhang, Yiqian Zhang, Xiaolin Meng, Tuoyu Cao, Xin Liu, Zhijin Fan, Hongcheng Shi

**Affiliations:** ^1^ Department of Nuclear Medicine, Zhongshan Hospital Fudan University Shanghai China; ^2^ United Imaging Healthcare Co., Ltd Shanghai China; ^3^ Center for Certification an Evaluation Shanghai Food and Drug Administration Shanghai China

**Keywords:** comfort survey, image quality, PET/MR, work flow

## Abstract

**Background:**

PET/MR is transferring from a powerful scientific research tool to an imaging modality in clinical routine practice. Whole body PET/MR screening usually takes 30–50 minutes to finish, during which a few factors might induce patient discomfort and further cause degraded image quality. The aim of this report is to investigate the patients' perception of the imaging procedure and its correlation with image quality.

**Methods:**

One hundred and twenty patients (63 males and 57 females, average age = 51.3 years, range 22–70 years) who had been diagnosed with cancer or had previous history of cancer were recruited and scanned with a simultaneous PET/MR system. A questionnaire was given to all patients retrospectively after the PET/MR scan, which has nine questions to assess patients' feeling of the scan on a Likert scale scoring system (1–5, 1 as most satisfied). All PET/MR images were also visually examined by two experts independently to evaluate the quality of the images. Six body locations were assessed and each location was evaluated also with a Likert scale scoring system (1–5, 5 as the best quality). Mann–Whitney U­test was used for statistical analysis to check if there is significant correlation between image quality and patient perceptions.

**Results:**

With a total of 120 patients, 118 questionnaires were filled and returned for analysis. The patients’ characteristics were summarized in Table 4. The statistics of the patients’ perception in the questionnaire were illustrated in Tables 5–7. Statistical significant correlations were found between MR image quality and patients’ characteristics/perception.

**Conclusion:**

Our results show that PET/MR scanning is generally safe and comfortable for most of the patients. Statistical analysis does not support the hypothesis that bad patient’s perception leads to degraded image quality.

## 1 BACKGROUND

There has been a rapid growth of interest in whole body simultaneous PET/MR scan in the past few years. And PET/MR is transferring from a powerful scientific research tool to an imaging modality in clinical routine practice.^1^ It has been demonstrated that PET/MR has a great potential in the area of neurology and oncology, such as Alzheimer's and Parkinson's Diseases (AD/PD), breast cancer, prostate cancer, colorectal carcinoma, melanoma, gynecological cancers, and brain tumors.^2^, ^3^, ^4^, ^5^, ^6^, ^7^ One of the major drawbacks of PET/MR imaging compared with PET/CT is the longer duration of scanning. Whole body PET/MR screening usually takes 30–50 minutes to finish, mainly due to the long scan time of MRI.^8^ During the long process, a few factors might induce patient discomfort, such as acoustic noise, local heating from RF energy, and pressure from the MR surface coil. Patients who are sensitive to these factors may disrupt the examination or move so much that image quality is severely degraded. Thus it is important to know the patients’ perception of the imaging procedure.

A few previous studies have evaluated the tolerance of patients during imaging examinations. Sparrow et al. compared the patients’ satisfaction and tolerance of MR and SPECT and found that more patients prefer MRI than SPECT with respect to tolerance and satisfaction during examinations.^9^ By using Likert scale, Shortman et al. compared PET/MR with PET/CT for the psychological burden before examinations, and found previous scanning experiences and communication with patients prior to and during PET/MRI improved patient satisfaction.^10^ Similar findings were also found in a study conducted by Acuff et al. Furthermore, improved patient satisfaction may have a positive effect on imaging, because it can reduce involuntary motion.^11^

PET/MR examination adopts all the technical challenges of a whole body MRI scan, including claustrophobia, physical discomfort, noise, scan duration, as well as the challenge of coping with emotions elicited during the scan such as fear/panic and isolation. In addition, subjects need to do preparations for PET imaging, include fasting, intake of water, administration of FDG, avoid motion, and unnecessary talking, this will further increase the stress and discomfort of the subjects during the examination. To the best of our knowledge, there has been no previous report on either patient comfort during whole body simultaneous PET/MR scans, or its correlation with diagnostic image quality. The aim of this paper is to report our survey results of the subjective patient response to varies factors during PET/MR scans and the impact of these factors on the final image quality. We hope this study can provide a guideline for designing a simultaneous PET/MR scan protocol.

## 2 MATERIAL AND METHODS

### 2.1 Patients

One hundred and twenty patients (63 males and 57 females, average age = 51.3 years, range 22–70 years) who had been diagnosed with cancer or had the previous history of cancer were recruited and scanned with a simultaneous PET/MR system. This study was approved by the institutional review board (IRB)/Ethics Committee of Zhongshan Hospital. All patients gave written informed consent. Inclusion and exclusion criteria were illustrated in Table 1.

**Table 1:** Inclusion and exclusion criteria for patient selection.

Inclusion criteria	Exclusion criteria
Age between 18 and 70 years old	Pregnancy
Agree to participate in this study and sign an informed consent form	With diabetes or with blood glucose levels greater than 182 mg/dL (10 mmol/L)
Able to understand mandarin and maintain good compliance	Allergic to PET imaging agents
Diagnosed with cancer or have a family history of cancer	Had Strenuous exercise within 24 hours before the PET/MR examination;
Has agreement from the referring physician	In a critical condition and require life support systems
	With epilepsy or other mental ills
	With other MRI contradictions

### 2.2 PET/MRI protocol and work flow

**Figure 1:**
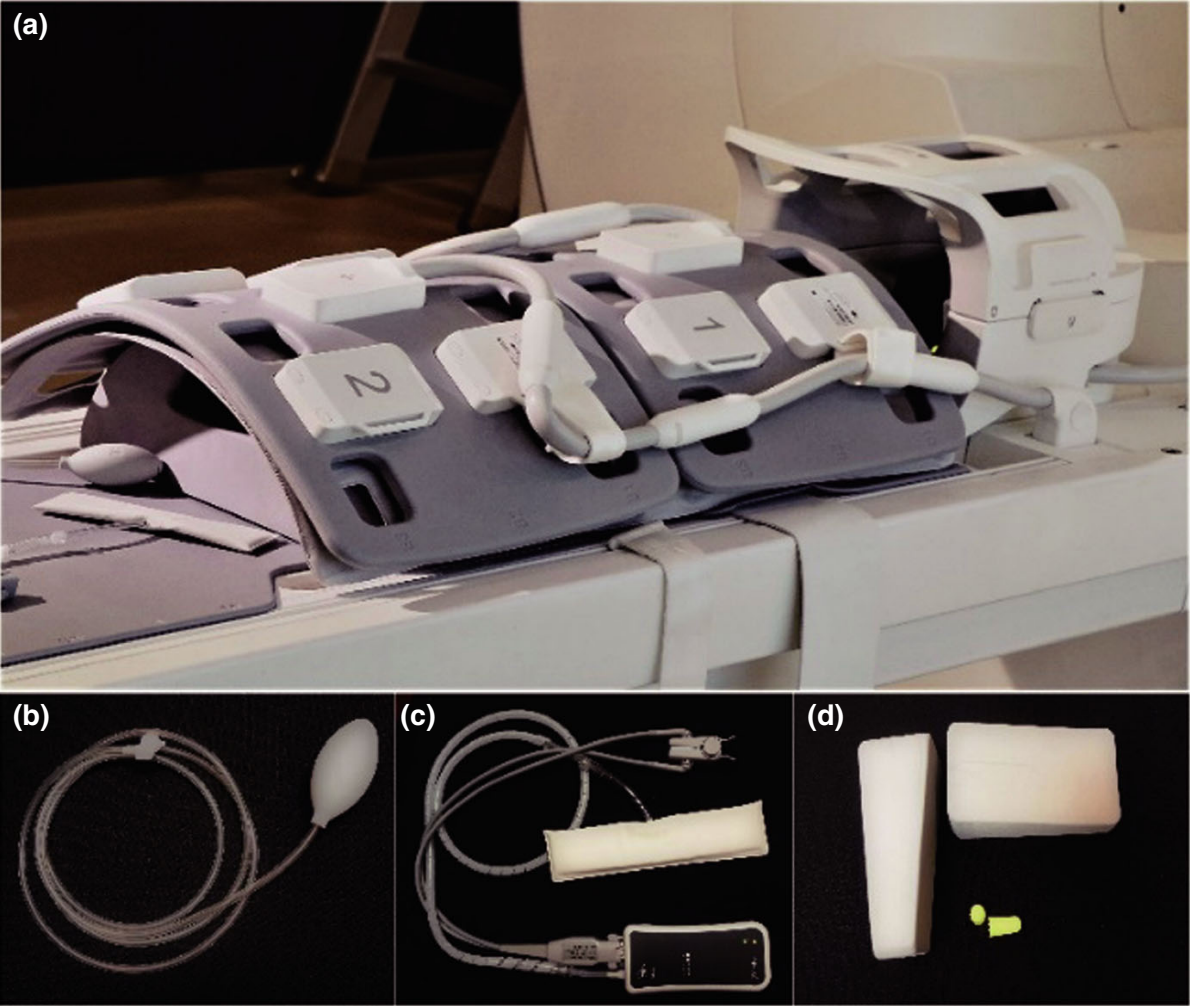
Coils and accessories, (a) dedicated PET/MRI whole‐body coil and head coil, (b) alarm ball, (c) vital signs monitor, and (d) wedge pad and earplugs.

Simultaneous whole‐body PET/MR acquisition was performed with an uPMR790 HD TOF PET/MR (United Imaging Healthcare, Shanghai, China). Patients were covered in a dedicated PET/MRI whole‐body coil and a head coil. Earplugs were given to all patients to mitigate the effect of noise from MRI gradient pulsing and the head is fixed using a wedge pad to minimize head motion during the scan. Vital signal monitoring (VSM) devices were used to monitor the respiration and heart rate of the patients, based on which the technician could communicate with the patient to ensure the patient was under a comfortable state for data acquisition (Figure 1). The PET/MR imaging protocol is shown in Table 2.

**Table 2:** MR protocol used in PET/MR examination.

SEQUENCE	TR	TE	THK	GAP	FOV	MATRIX
scout_wb	8.8	4.5	10	40	50 × 136	874 × 320
wb_pet_general						
ac_wfi	4.6	3.2	2.4	0	500 × 350	206 × 144
I_t2_ssfse_cor	1300	94.2	6	1.8	450 × 350	393 × 408
I_stir_fse_cor	4899	50	6	1.8	280 × 450	223 × 480
ac_wfi_bh	4.6	3.2	2.4	0	500 × 350	206 × 144
II_t2_ssfse_cor	1300	94.2	6	1.8	450 × 350	393 × 408
II_t2_fse_tra_fs_trig	3916	88.4	6	1.2	300 × 380	252 × 456
II_epi_dwi_tra_trig	3529	67.9	6	1.2	300 × 380	202 × 256
ac_wfi_bh	4.6	3.2	2.4	0	500 × 350	206 × 144
III_t2_ssfse_cor	1300	94.2	6	1.8	450 × 350	393 × 408
III_t2_arms_tra_spair	2278	100.5	6	1.2	380 × 380	288 × 288
ac_wfi	4.6	3.2	2.4	0	500 × 350	206 × 144
IV_t2_ssfse_cor	1300	94.2	6	1.8	450 × 350	393 × 408
IV_t2_fse_tra_spair	3968	90	6	1.2	300 × 380	337 × 504
Optional						
neck_stir_arms_tra	5161	100	6	1.2	380 × 380	576 × 576
thorax_ssfse_tra	1200	107.6	6	1.2	300 × 380	318 × 504
pelvis_t2_fse_tra_spair	3968	90	6	1.2	300 × 380	337 × 504
pelvis_epi_dwi_tra	3127	69.6	6	1.2	300 × 380	176 × 224

### 2.3 Patient comfort survey

A questionnaire^12^ was given to all patients after the PET/MR scan. The questionnaire has nine questions (Table 3) to assess patients’ perception of the scan on a Likert scale scoring system (1‐5, 1 as the most satisfied).

**Table 3:** Patient perception questionnaire.

1. Please rate the preparation and information before this examination
2. Please rate your degree of anxiety before this examination
3. Please rate the overall comfort level of this examination
4. Please rate satisfaction level of the scanning time of this examination
5. Please rate satisfaction level of the heat during this examination
6. Please rate satisfaction level of the acoustic noise during the examination
7. Please rate satisfaction level of the coil heavy covered on your body
8. Please rate satisfaction level of the peripheral nerve stimulation effect
9. Please rate your overall satisfaction level with this test

### 2.4 Image quality evaluation

The PET/MR images for all patients were evaluated independently by two radiologists who are qualified of reading both MRI and PET images. Six body locations were assessed and each location was evaluated on PET Image quality, MR image quality and error of fusion with a Likert scale scoring system (1‐5, 5 as the best quality).

### 2.5 Statistical analysis

Three categories of 14 factors in total were analyzed in this study and their correlation with image qualities was also studied. The first category includes patients’ characteristics such as patients’ gender, age, weight, and height. To explore the correlation between image quality and these factors, patients were divided into two groups based on each factor and median value for each factor was used as the boundary between two groups. The second category contains all the nine questionnaire items, and for each item patients were divided into two groups, group A and group B, in a manner that the number of patients in each group should be as close as possible. The third category is the scan time, and the patients were also divided based on the median value of the scan time. Since Likert scale score is considered as ordinal data, a nonparametric test (Mann‐Whitney U test) was used to check if there is a significant difference between the image qualities of each of six body locations of the patients from these two groups. The value of image quality was obtained through calculating the average of the assessments of the two radiologists.

To further study the patients’ tendency of giving scores, the patients were also grouped based on one simple criteria: whether he/she has gave a score of 4 or 5 to any of these nine questions. Patients giving at least one score of 4 or 5 were put into group C while the rest were put into group D. And the average score of these two groups on each of the nine questions were calculated. The difference of patents’ characteristics (gender, weight, age, and height) between group C and D were also analyzed.

All the statistical analysis was performed in R‐software environment(R Foundation for Statistical Computing, Vienna, Austria).

## 3 RESULTS

With a total of 120 patients, 118 questionnaires were filled and returned for analysis. The patients’ characteristics were summarized in Table 4. The statistics of the patients’ perception in the questionnaire were illustrated in Tables 5, 6, 7. The average overall perception among the patients is 1.36 (1‐5, 1 is the most satisfied) which indicated that PET/MR scanning is generally safe and comfortable for most of the patients.

**Table 4:** Patient characteristics.

Characteristics	Statistics
Age (year)	
Median	55
Mean ± STD	52.16 ± 12.82
Range	22‐70
Gender	
Male	61
Female	57
Height (cm)	
Mean ± STD	165.75 ± 8.75
Weight (kg)	
Mean	63
BMI (kg/m^2^)	
Mean ± STD	22.90 ± 2.97
GLU (mmol/L)	
Mean ± STD	4.89 ± 0.87

**Table 5:** Patients’ comfort survey result.

	1	2	3	4	5	Mean	Median
Satisfaction level of preparation	89 (75.42%)	28 (23.73%)	1 (0.85%)	0	0	1.2542	1
Anxiety level before scan	75 (63.56%)	41 (34.75%)	2 (1.69%)	0	0	1.3814	1
Overall comfort level	65 (55.08%)	41 (34.75%)	10 (8.47%)	2 (1.69%)	0	1.5678	1
Satisfaction level on scan duration	51 (43.22%)	36 (30.51%)	21 (17.8%)	9 (7.63%)	1 (0.85%)	1.9237	2
Feeling on heating	21 (17.8%)	44 (37.29%)	29 (24.58%)	17 (14.41%)	7 (5.93%)	2.5339	2
Feeling on acoustic noise	19 (16.42%)	40 (34.19%)	45 (38.46%)	8 (6.84%)	5 (4.27%)	2.4872	2
Feeling on surface coil pressure	65 (55.09%)	36 (30.51%)	9 (7.63%)	6 (5.08%)	2 (1.69%)	1.7627	1
Feeling on peripheral neural stimulation	96 (81.36%)	18 (15.25%)	3 (2.54%)	0	1 (0.85%)	1.2373	1
Overall perception	81 (68.64%)	34 (28.81%)	1 (0.85%)	2 (1.69%)	0	1.3559	1

Note

Each table item shows the frequency of the answer for each question (relative frequency in percentage is shown in the bracket). Mean and median values were also shown in the table.

**Table 6:** Average image quality scores for group A.

	Head	Neck	Thorax	Abdomen	Pelvis	Lower extremity
Satisfaction level of preparation	4.4101	4.1180	3.8708	4.0562	4.0618	4.2247
Anxiety level before scan	4.4667	4.1333	3.9000	4.1067	4.0467	4.1733
Overall comfort level	4.4385	4.1308	3.8615	4.1077	4.0462	4.1615
Satisfaction level on scan duration	4.4118	4.1471	3.9020	4.0980	4.0098	4.1275
Feeling on heating	4.3385	4.1077	3.8615	4.0077	4.0462	4.1615
Feeling on acoustic noise	4.4000	4.1083	3.8333	4.0750	4.0917	4.2417
Feeling on surface coil pressure	4.4141	4.1250	3.8698	4.1042	4.0833	4.2031
Feeling on peripheral neural stimulation	4.4635	4.1250	3.8698	4.1042	4.0833	4.2031
Overall perception	4.4506	4.1420	3.8519	4.0864	4.0679	4.2160

**Table 7:** Average image quality scores for group B.

	Head	Neck	Thorax	Abdomen	Pelvis	Lower extremity
Satisfaction level of preparation	4.6207	4.2241	3.8966	4.3276	4.2414	4.4138
Anxiety level before scan	4.4535	4.1628	3.8372	4.1512	4.2093	4.4419
Overall comfort level	4.4906	4.1604	3.8962	4.1415	4.1792	4.4057
Satisfaction level on scan duration	4.5000	4.1418	3.8582	4.1418	4.1791	4.3806
Feeling on heating	4.6132	4.1887	3.8962	4.2642	4.1792	4.4057
Feeling on acoustic noise	4.5259	4.1810	3.9224	4.1724	4.1207	4.3017
Feeling on surface coil pressure	4.5185	4.1667	3.8426	4.2130	4.1759	4.3611
Feeling on peripheral neural stimulation	4.4545	4.2273	3.9091	4.2045	4.2045	4.5682
Overall perception	4.4865	4.1486	3.9324	4.2027	4.2027	4.3919

Among nine questions in the questionnaire, patients complained most about feeling on heating (Average: 2.53) and feeling on acoustic noise (Average: 2.48), which indicates that heating and acoustic noise during the scan procedure might have a great influence on patients’ comfort level.

### 3.1 Patients’ characteristics vs image quality

Correlation between MRI image quality and gender as well as body weight were studied. The statistical test revealed that gender has an impact on MR image quality on thorax and lower extremity (*P* = 0.025 and *P* = 0.038, respectively). The mean value of MRI image quality on thorax for man and woman are 3.81 and 3.73, respectively. And mean value of MRI image quality on lower extremity for man and woman are 4.62 and 4.20, respectively. These results revealed that MRI image quality in man on these two locations were better than woman. Moreover, body weight can also affect MR image quality on thorax (*P* = 0.037) that there is a significant difference in MR image quality between the patient group with body weight larger than 62 kg and those with body weight smaller than 62 kg (image quality score: 3.81 and 3.94, respectively). However, PET image quality and image fusion quality has no significant correlation with these factors.

### 3.2 Patient comfort survey vs image quality

Based on the characteristics of all the answers in the questionnaire, for question 5 and 6, group A has all the patients with the answer of 1 and 2, while the group B has the rest of the patients. For the other seven questions, group A has the patients with the answer of 1 while group B has the answer of 2 to 5.

**Figure 2:**
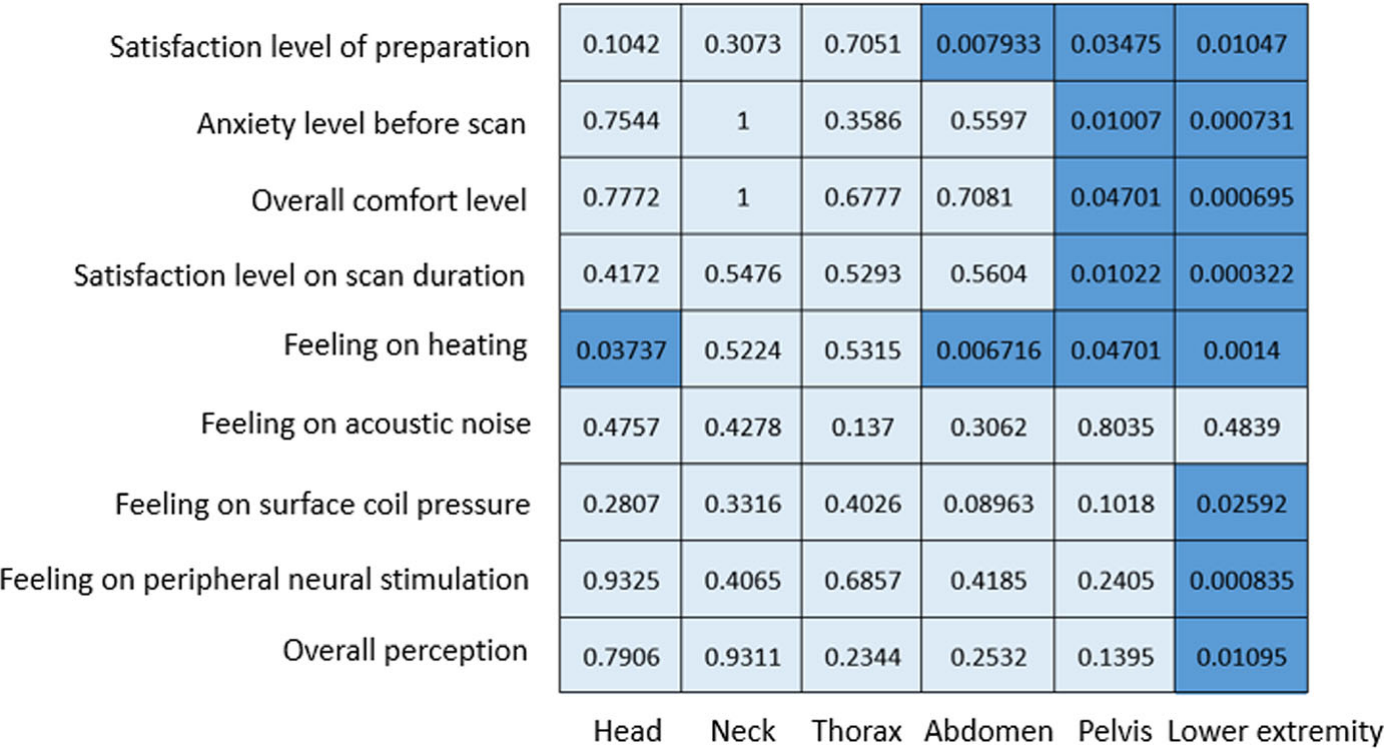
*P* value of the statistical test of the correlation between MR image quality and questionnaire items. Items with significant difference are highlighted (*P* < 0.05).

No significant correlation was observed between these questionnaire items and PET image quality or image fusion quality. The result between these factors and MR image quality is summarized in Figure 2. In general, heating has the most significant impact on MR image quality. While MR image quality of lower extremity is most sensitive to patient feelings during the scan compared to other body locations.

The average image quality score for each body position in each group is showing in Table 8. For example the first number (upper left) in Tables 9 and 10 means that the average image quality value from all patients who gave answer of 1 to the question of “satisfaction level of preparation” is 4.41. The average image quality score of group A is almost always lower than those of group B, while for pairs showing statistically significant difference in Mann‐Whitney U test, group A always has a lower average score.

**Table 8:** Average scores of group A and B in all nine questions.

	Group A	Group B
Satisfaction level of preparation	1.4440	1.3333
Anxiety level before scan	1.6667	1.7778
Overall comfort level	1.8889	1.4444
Satisfaction level on scan duration	2.5556	2.0000
Feeling on heating	4.1111	2.5556
Feeling on acoustic noise	3.0000	2.2500
Feeling on surface coil pressure	2.6667	1.5556
Feeling on peripheral neural stimulation	1.7778	1.0000
Overall perception	1.6667	1.4444

**Table 9:** Average image quality scores for group A.

	Head	Neck	Thorax	Abdomen	Pelvis	Lower extremity
Satisfaction level of preparation	4.4101	4.1180	3.8708	4.0562	4.0618	4.2247
Anxiety level before scan	4.4667	4.1333	3.9000	4.1067	4.0467	4.1733
Overall comfort level	4.4385	4.1308	3.8615	4.1077	4.0462	4.1615
Satisfaction level on scan duration	4.4118	4.1471	3.9020	4.0980	4.0098	4.1275
Feeling on heating	4.3385	4.1077	3.8615	4.0077	4.0462	4.1615
Feeling on acoustic noise	4.4000	4.1083	3.8333	4.0750	4.0917	4.2417
Feeling on surface coil pressure	4.4141	4.1250	3.8698	4.1042	4.0833	4.2031
Feeling on peripheral neural stimulation	4.4635	4.1250	3.8698	4.1042	4.0833	4.2031
Overall perception	4.4506	4.1420	3.8519	4.0864	4.0679	4.2160

**Table 10:** Average image quality scores for group B.

	Head	Neck	Thorax	Abdomen	Pelvis	Lower extremity
Satisfaction level of preparation	4.6207	4.2241	3.8966	4.3276	4.2414	4.4138
Anxiety level before scan	4.4535	4.1628	3.8372	4.1512	4.2093	4.4419
Overall comfort level	4.4906	4.1604	3.8962	4.1415	4.1792	4.4057
Satisfaction level on scan duration	4.5000	4.1418	3.8582	4.1418	4.1791	4.3806
Feeling on heating	4.6132	4.1887	3.8962	4.2642	4.1792	4.4057
Feeling on acoustic noise	4.5259	4.1810	3.9224	4.1724	4.1207	4.3017
Feeling on surface coil pressure	4.5185	4.1667	3.8426	4.2130	4.1759	4.3611
Feeling on peripheral neural stimulation	4.4545	4.2273	3.9091	4.2045	4.2045	4.5682
Overall perception	4.4865	4.1486	3.9324	4.2027	4.2027	4.3919

The regroup resulted in 39 patients in group C and 79 patients in group B. The average scores for each of the nine questions from group C and D were shown in Table 11. Furthermore, no significant difference was observed between group C and D in terms of patient characteristics.

**Table 11:** Average scores of group C and D in all nine questions.

	Group C	Group D
Satisfaction level of preparation	1.44	1.33
Anxiety level before scan	1.67	1.78
Overall comfort level	1.89	1.44
Satisfaction level on scan duration	2.56	2.00
Feeling on heating	4.11	2.56
Feeling on acoustic noise	3.00	2.25
Feeling on surface coil pressure	2.67	1.56
Feeling on peripheral neural stimulation	1.78	1.00
Overall perception	1.67	1.44

### 3.3 Scan time vs image quality

The mean scanning time of the patients are 52.8 minutes with standard deviation of 8.5 minutes. The study shows that scan time is another important factor affecting MR image quality of the neck location (*P* = 2.2E‐6). The MR image quality is getting significantly worse when scan time is beyond 52 minutes (Mean value: 4.22 and 4.07 for scan time below and beyond 52 minutes, respectively.).

## 4 DISCUSSION

Many previous literatures have been published on patients’ perception during MRI scans, especially on the topic of claustrophobia.^13^ However, there is few quantitative research on the patient’s perception and its influence on image quality. Thus this survey might provide valuable information for PET/MR manufactures and PET/MR technicians to improve the quality of patient care. The results of the study showed that the average score of patient overall perception is 1.36, indicating that most patients can tolerate PET/MR examination. Among all the factors, the scores regarding heating and noise were the highest. Even earplugs were used during all the scans, the strong noise caused by MRI gradient pulsing is still a major factor affecting patient’s comfort. A common way to mitigate the noise effect in a MRI scan is through using less gradient intensive sequences. However, this is not a practical option for PET/MR, because PET/MR scans are usually much longer than a regular MRI scan and compromise in gradient intensity would require even longer scan duration. Better sound isolation and force balanced gradient design should be a more important concern in PET/MR systems than in MRI systems. Patients’ feeling on heating is another common complaint during PET/MR scans. The usage of a large surface coil covering most of the patient’s body might be an important factor because it limits the heat dissipation. Another reason is that RF intensive MR sequences was included in our protocol resulting in more heat production.

**Figure 3:**
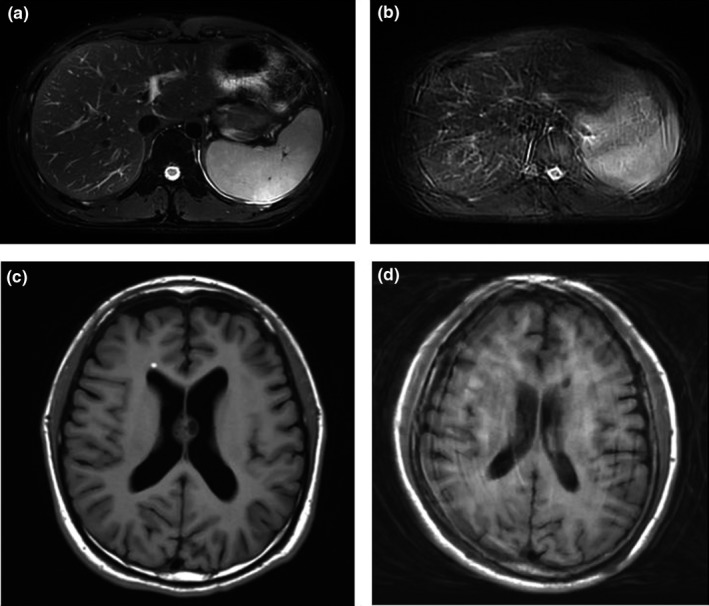
(a) ARMS abdominal image with no motion artifact (b) ARMS abdominal image with strong motion artifact (c) T1W head image with no motion artifact (d) T1W head image with strong motion artifact.

In this study, image quality scores were obtained by a subjective metric, that is, evaluation from two radiologists. The human visual system is the gold standard in image quality evaluation.^14^ However, subjective evaluation by human observers suffers from high intra‐reader and inter‐reader variability. On the other hand, objective image quality evaluation^15^ has been an active research area mainly driven by the computer vision community to solve tasks such as storage, compression and transmission. Depending on the availability of a reference image, objective image quality evaluation has three subcategories, including full reference image quality assessment (FR‐IQA), reduced reference image quality assessment (RR‐IQA)^16^ and no reference image quality assessment (NR‐IQA).^17^, ^18^, ^19^, ^20^, ^21^ There are a few widely accepted FR‐IQA techniques, such as Mean Squared Error (MSE),^22^ Peak Signal‐to‐Noise Ratio (PSNR),^23^ and Structural similarity Index (SSIM).^24^ However, only NR‐IQA is applicable for the purpose of evaluating medical images in this study. In general, NR‐IQA is very challenging because no pristine image is available to compare with and the evaluation is highly application specific.^25^ In our study, the primary factor that affects image quality is motion artifact. And motion artifact could have complex appearances because it is mixed with anatomical structures, which makes it difficult to quantify with standard quantitative metrics. Examples of MR motion artifact are shown in Figure 3 below.

In general, the factors studied in this research have very little effect on PET image quality or the image fusion accuracy. This might due to the fact that the mechanism of PET imaging is very different compared to MRI. A PET image is an average over a duration of a few minutes so a few seconds of corrupted data has little effect on the overall image quality. While MRI has a more complicated mechanism of generating contrast and a short motion could cause strong artifact in MR images if the motion happens during the data acquisition near the center of k‐space. Another reason might be that it is harder to visually evaluate the quality of a PET image than a MR image because PET images have very limited structure information. Moreover, gating was used during the bed station of thorax so the respiratory motion artifact was minimized. One example is curvilinear cold artifact which is commonly seen in PET/CT images because of respiration mismatch between PET images and CT attenuation correction.

According to the statistical test, heavy body weight has a negative effect on MR image quality. This finding is consistent with the situation in standalone MRI imaging. MR images of bigger patients usually suffer from worse signal to noise ratio and it is more likely to have image artifacts when the subject is closer to the edge of the field of view due to the deterioration of B0/B1 field homogeneity and gradient linearity. Another possible reason is that a heavier patient might feel more constricted in the bore of the system considering the bore size of the PET/MR system is only 60 cm. Even though it is more technically challenging to make a wide bore PET/MR system than a standalone MRI system due to the extra spaced taken by the PET ring, it would be helpful to extend the bore size for a better patient perception.

Our study also shows that there are some correlations between the questionnaire items and the MR image quality. Initially we hypothesized that a higher comfort level would lead to better image quality under the rational that patients with higher comfort level would be more cooperative. However, further analysis of the data shows completely opposite results and the results are consistent among all entries with statistically significant difference. The fact that a higher comfort level leads to worse image quality is counterintuitive. Moreover, further analysis shows that patients giving higher scores for one question tend to give higher scores for most questions (with the only exception being anxiety level before scan, which is not for access the perception during scans). One possible explanation for this interesting result is that patients giving negative feedback are those who maintained conscious during the whole scan process. According to our record, about 30% of the patients had fallen into sleep shortly after the scan begins. And when a patient losses consciousness, he/she would not able to maintain a regular breath pattern or be responsive to the scan instructions. A sleepy patient can also have unconscious motion during the scan. All of these factors could induce image artifacts. This finding suggests that sometimes it is not a good sign that a patient is feeling “too comfortable” during a PET/MR scan. It is important to educate the patient so that they can maintain their body control during the entire scan process. And the operator should monitor the statues of the patient constantly to make sure the patient is responsive and cooperative. Furthermore, the in‐bore environment such as ventilation and lighting should be adjusted accordingly.

Moreover, overall scan time is another important factor that could affect image quality. All patients in this study were scanned with head‐in‐first supine body position and scanned from bottom to top. This is designed to minimize the effect of the continuous bladder expanding. Thus head and neck is the last scan location for each patients and the data shows that its image quality could suffer from the long scan duration. This is a strong evidence that it is crucial to optimize the clinical work flow to minimize the scan time.

## 5 CONCLUSIONS

Patient survey results were collected from 118 patients after PET/MR scans. Image quality of these patients was evaluated by two radiologists. Statistical analysis was performed to study the correlation between image qualities and varies of factors. Our results show that PET/MR scanning is generally safe and comfortable for most of the patients. Statistical analysis does not support the hypothesis that bad patient’s perception leads to degraded image quality. And maintaining control of the patient’s during the scan might be crucial during whole body PET/MR examination.

## AUTHORS’ CONTRIBUTIONS

HCS created the design of the study and analysis and supervised the project. SGC created the design of the study collected and analyzed the data, and drafted the manuscript. HPC contributed to statistical analysis and manuscript revision. GYC, ZZ, CTY, and YYZ participated in the design of the study and analysis and contributed to the manuscript revision and editing. All authors read and approved the final manuscript.

## CONSENT FOR PUBLICATION

All authors read the manuscript and consented for its publication.

## CONFLICT OF INTERESTS

The authors declare that they have no competing interests.

## ETHICS APPROVAL AND CONSENT TO PARTICIPATE

All procedures performed in studies involving human tissue were in accordance with the ethical standards of the institutional and/or national research committee and with the principles of the 1964 Declaration of Helsinki and its later amendments or comparable ethical standards.

## DATA AVAILABILITY STATEMENT

Please contact the author for data requests.
